# The Metric System

**Published:** 1895-08-24

**Authors:** 


### The Metric System.

Two announcements have recently been made, which draw renewed attention to the subject of metric measurements; one a statement as to the use of the metric system, at least as an alternative, in the new Pharmacopœia, the other the report of the Select Committee on Weights and Measures, recommending the legalisation of this system for all purposes. Just a hundred years ago, in the year 1795, the metric system of weights and measures, which is now in universal use throughout the world among scientific workers, was proposed in France. The history of France during the last ten years of the last century is so crowded with the striking events of the great Revolution that small affairs are apt to be overlooked. The red bonnet, the guillotine, the abolition of privilege, the death of kings, the reign of terror, the rise of Napoleon—these things figure so largely in that wonderful ten years' history as to put into the background the less startling results of that search for novelty and change which was so marked a characteristic of the period; but however necessary the more salient facts may be for the purposes of the historian in painting a picture of the time, it is none the less interesting to be reminded how the higher life of science went on through these troublous days. In 1790, the year after the destruction of the Bastille, a proposal was made by the French Government to that of Great Britain for a meeting to take place between delegates of the Academy of Sciences and the Royal Society of London to determine the length of a pendulum which would oscillate in a second of time, with the view of making this the unit of a system of measures which might become universal. The British Government, however, did not favourably entertain the suggestion, and the conference did not take place. The French Government then being anxious that a standard should be arrived at, appointed a commission of physicists and mathematicians to choose the one best fitted for the purpose from among the following three alternative suggestions, viz., the length of the second's pendulum, the fourth part of the equator, and the fourth part of the meridian. The decision of this commission fell upon the last, and it was resolved that the one-ten-millionth part of a quadrant of the meridian, *i.e.*, of the distance from the equator to the pole, should be the basis of the new system, and that this fraction of the meridian should be described as a "metre."

The next step was the actual measurement of a defined portion of a meridian. In 1792 this work was commenced by Delambre and Méchain at the instance of the Government, the line taken running right across France from Dunkerque to Barcelona; and when one recalls the events of that year, the march of the mob upon the Tuileries, the slaughter of the Swiss guards, the massacres in Paris, the breaking open of the prisons, and in external politics the coalition of the Great Powers against France and the commencement of the great war, one cannot but express admiration for the versatility of a people who at such a moment could busy themselves over so abstruse a matter as the establishment of a universal standard of measurement, and could set themselves the task of measuring a meridian for the sake of making this standard theoretically perfect. This quiet, peaceful, but laborious process of measurement went on through the frightful days of the reign of terror; the result was then submitted to a commission partly French and partly foreign, and on April 7th, 1795, the metre was by law made the unit of length and the base of the metric system. It was not, however, till November 2nd, 1801, that the new system was finally accepted as the only legal one.

Certain difficulties even then arose in its adoption, and an attempt was made to accommodate the old measures to the new; but on July 4th, 1837, it was finally decreed that the metric system should be used in all business transactions.

Thus was finally established, if finality can be asserted for anything, a system which arose in the very froth and turmoil of revolution and took form and shape during a time when fixity or permanence seemed assured to nothing. Its inception was in the very year when the old régime was abolished and France was declared a limited monarchy, and before the system was finally established a Republic had been set up, and both king and queen had been beheaded; another King, Louis XVII., had died in prison, France had been made an Empire, with Napoleon for its Emperor; this had been cast down and the old dynasty had been restored, Napoleon had upset it and again seized the throne, in his turn to be banished within a year; the direct line had been once more restored, but had fallen by the abdication of Charles X., and, lastly, Louis Phillipe had accepted the throne. Through all these changes and revolutions a useful measure of reform had worked its way, a fact which may well suggest how largely the evolution of domestic legislation is independent of that race for power of which, in the eyes of many politicians, politics consist. Meanwhile in England the question of introducing the metric system is by no means a new thing. It was brought before Parliament by Sir John Wrottesley so early as the year 1824; a commission of inquiry was appointed in 1838 and another in 1843, and both reported strongly in favour of the change; a committee of the House of Commons reported to the same effect in 1853; another commission was appointed in 1855, and published evidence, but expressed no opinion; an Act "to render permissive the use of the metric system of weights and measures" was passed in 1864, but was repealed by the Weights and Measures Act, 1878; a Bill for the compulsory adoption of the metric system was rejected by the Commons in 1871, and another proposal in its favour, introduced by Mr. Ashton Dilke, was negatived by the Commons in 1881; and now we have the report of a Select Committee recommending its legalisation for all purposes.

Whatever the fate of this proposal, it is clear that in regard to medicine there is nothing to be gained by adhesion to the complicated arrangement—system it cannot be called—which is at present in operation, which separates medicine from all other sciences, and renders the prescriptions of English physicians incomprehensible to the outer world.

